# Integrative Analysis for Elucidating Transcriptomics Landscapes of Systemic Lupus Erythematosus

**DOI:** 10.3389/fgene.2021.782005

**Published:** 2021-11-04

**Authors:** Haihong Zhang, Yanli Wang, Jinghui Feng, Shuya Wang, Yan Wang, Weisi Kong, Zhiyi Zhang

**Affiliations:** ^1^ Department of Rheumatology and Immunology, The First Affiliated Hospital of Harbin Medical University, Harbin, China; ^2^ Department of Gerontology, The First Affiliated Hospital of Harbin Medical University, Harbin, China

**Keywords:** systemic lupus erythematosus, differential expression analysis, gene functional enrichment analysis, RNA-seq, protein-protein interaction

## Abstract

Systemic lupus erythematosus (SLE) is a complex and heterogeneous autoimmune disease that the immune system attacks healthy cells and tissues. SLE is difficult to get a correct and timely diagnosis, which makes its morbidity and mortality rate very high. The pathogenesis of SLE remains to be elucidated. To clarify the potential pathogenic mechanism of SLE, we performed an integrated analysis of two RNA-seq datasets of SLE. Differential expression analysis revealed that there were 4,713 and 2,473 differentially expressed genes, respectively, most of which were up-regulated. After integrating differentially expressed genes, we identified 790 common differentially expressed genes (DEGs). Gene functional enrichment analysis was performed and found that common differentially expressed genes were significantly enriched in some important immune-related biological processes and pathways. Our analysis provides new insights into a better understanding of the pathogenic mechanisms and potential candidate markers for systemic lupus erythematosus.

## Introduction

Systemic lupus erythematosus is a chronic autoimmune disease (Beccastrini et al., 2013; Davies et al., 2021). Its clinical manifestations are heterogeneous and involve one or more organs such as skin, kidney, joints, and nervous system (Von Feldt, 1995; Adinolfi et al., 2016; Ronco et al., 2021). The latest data from the US Lupus Registry and published studies around the world can more accurately estimate the incidence and prevalence of SLE. It is estimated that the incidence of 23.2 cases per 100,000 people in North America is the highest in the world (Tsokos, 2011; Rees et al., 2017). SLE is a heterogeneous rheumatic systemic disease with extremely diverse clinical manifestations and diverse pathogenesis (Wu et al., 2021). In addition, it is one of the most varied diseases in its epidemiology and etiology, with different types of immune dysfunction (Oku and Atsumi, 2018). SLE patients’ immune system activation is characterized by exaggerated B cells and T cells responses (Tsokos, 2011). The health-related quality of life of SLE patients is significantly impaired (Di Battista et al., 2018). To obtain a better diagnosis and treatment method, it is necessary to explore the pathogenesis of SLE.

Since the successful application of high-throughput technology, it has been widely used in almost all biological research fields (Hess et al., 2020). With the development of high-throughput technology (Hess et al., 2020), biological research has been transformed from a single gene level to a full transcriptome level, which has greatly advanced many research fields in biology (Wang et al., 2009; McDermaid et al., 2019). Cheng. et al. based on the genome-wide expression data of peripheral blood mononuclear cells (PBMC) of SLE patients found a novel marker of SLE (Cheng et al., 2021). Jiang. et al. discovered a new type of lncRNA that plays an important role in the pathogenesis of SLE based on the whole transcriptome data of PBMC of SLE patients (Jiang et al., 2021). However, these studies were only conducted on a single dataset, and there was heterogeneity between different datasets. Therefore, through a comprehensive analysis of multiple datasets, more robust results will be obtained.

In this study, we conducted a systematic analysis of two gene expression datasets of SLE. First, differential expression analysis was performed to obtain differentially expressed genes (DEGs) in each dataset. To obtain robust results, we intersected the DEGs s of the two datasets. We found that 790 genes were differentially expressed in both datasets. Finally, gene function enrichment analysis showed that common DEGs were enriched in immune-related biological pathways. Overall, our research provided new insight into the molecular mechanism of SLE.

## Materials and Methods

### Datasets

“Systemic Lupus Erythematosus” and “RNA-seq” were used as the keywords for searching the GEO database. The gene expression datasets of PBMC from freshly isolated healthy controls and SLE patients were downloaded from the GEO database (GSE162828 and GSE169080), the platforms used were GPL24676, and GPL20795. GSE162828 included 10 samples of peripheral blood mononuclear cells and was divided into the SLE group (5 samples) and healthy controls group (5 samples). GSE169080 included seven samples of peripheral blood mononuclear cells and was divided into SLE group (4 samples) and healthy controls group (3 samples) (Clough and Barrett, 2016; Cheng et al., 2021; Jiang et al., 2021).

### Data Pre-processing and Identification of Differential Expressed Genes

R package DESeq2 (1.26.0) was used for the analysis of the original datasets (Love et al., 2014). |log FC| > 1 and p. adj <0.05 were defined as the cutoff values for further analysis of DEGs. Volcano and heatmap were constructed by R package ggplot2. Venn plot (http://bioinformatics.psb.ugent.be/webtools/Venn/) was used to draw the intersection of two databases.

### Analyzing of DEGs on Protein-Protein Interaction Network

Protein-protein interaction (PPI) network analysis helps to study the molecular mechanism of diseases from a systematic perspective and discover new drug targets (Wu et al., 2019). STRING (https://string-db.org/) is a database covering more than 5,000 organisms with known and predicted protein-protein interactions, providing direct (physical) and indirect (functional) associations (Szklarczyk et al., 2017). We used String (https://string-db.org/) to generate biological networks for proteins, and the results were analyzed by Cytoscape (Shannon et al., 2003; Szklarczyk et al., 2017).

### Gene Functional Enrichment Analysis

Gene Ontology (GO) is an ontology widely used in the field of bioinformatics, which covers three aspects of biology: biological process (BP), cellular component (CC), and molecular function (MF) (Thomas, 2017). Kyoto Encyclopedia of Genes and Genomes (KEGG) is a biological system advanced function and utility database based on molecular-level information from genome sequencing and other high-throughput experimental technologies (Kanehisa et al., 2017). In this study, R package clusterProfiler was used to perform GO functional annotation and KEGG pathway enrichment analysis for DEGs (Yu et al., 2012).

## Results

### Differentially Expressed Genes Between SLE Patients and Healthy Controls

**FIGURE 1:**
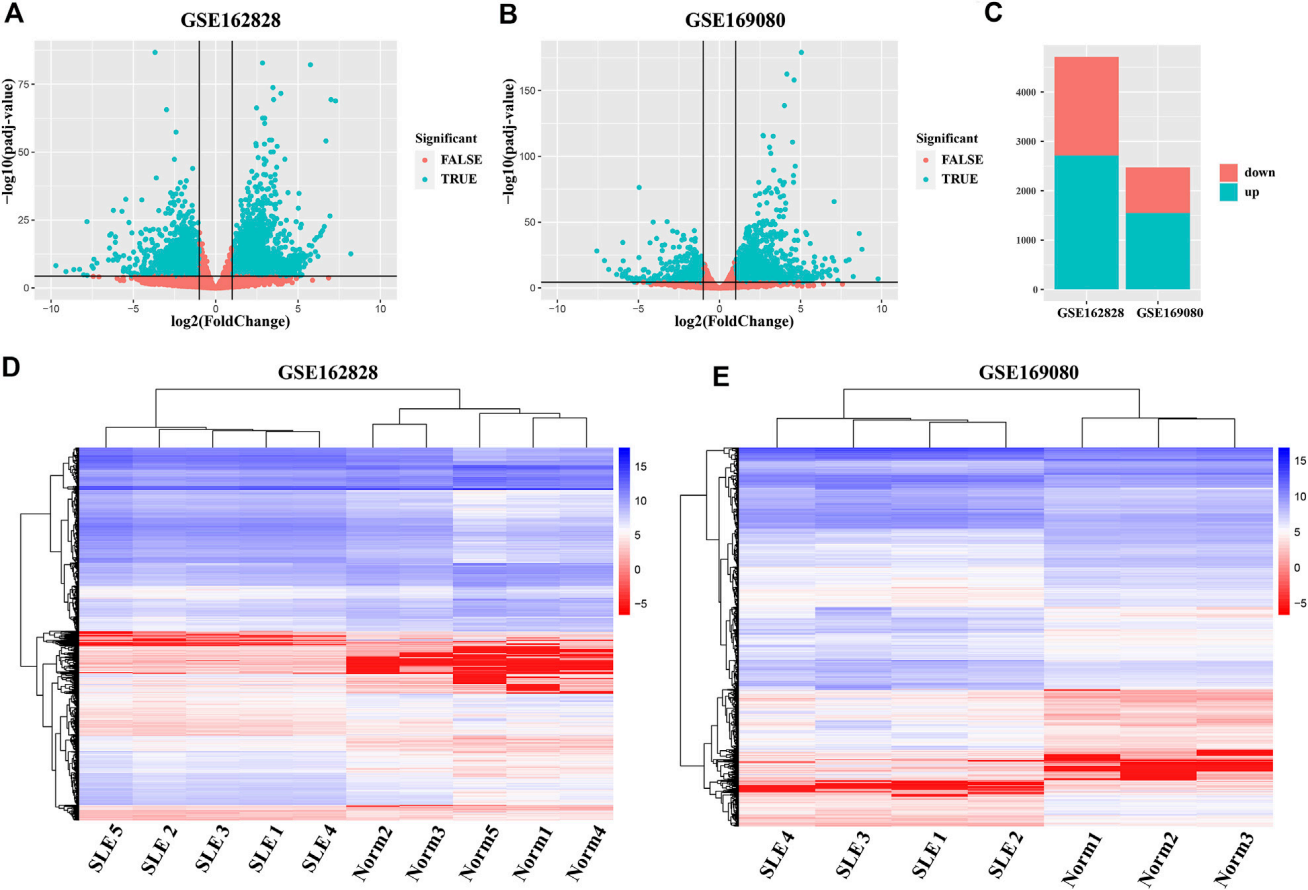
Analysis of differentially expressed genes (DEGs) between SLE patients and healthy controls. **(A,B)** The volcano plots exhibited the differentially expressed genes in SLE patients groups compared to healthy controls groups. Each dot in the figure represented one gene. The green dots indicated the differentially expressed genes, while the red dots denoted no significant difference. **(C)** Barplot showed the number of DEGs whose expression levels were up-regulated (green) and down-regulated (red) in the two datasets. **(D, E)** Hierarchy Clustering Analysis. Repeated samples are clustered together, indicating the repeatability of samples and the differences between samples.

To obtain abnormally expressed genes in SLE patients, we separately analyzed the differential expression of two GEO datasets (GSE162828 and GSE169080). As shown in Figure 1A, there were 4,713 DEGs, including 2,717 up-regulated and 1,996 down-regulated in the GSE162828 dataset. In the GSE169080 dataset, there were 2,473 DEGs, including 1,552 up-regulated and 921 down-regulated (Figure 1B). In both datasets, the number of up-regulated DEGs was more than the number of down-regulated DEGs (Figure 1C). In the GSE162828 dataset, the up-regulated DEGs accounted for 56.7% of all DEGs. At the same time, the up-regulated DEGs accounted for 62.8% of all DEGs in the GSE169080 dataset. The trends in the two datasets were roughly the same.

In addition, the heatmap showed that DEGs can group samples by sample type, namely SLE patients (SLE) and healthy controls (Norm) (Figures 1D,E). These genes were highly concordant within groups. The expression level of these genes between SLE patients and healthy controls exhibited a large difference in both databases.

### Identification of Common Differentially Expressed Genes by Integrated Analysis

Due to the heterogeneity between different datasets, the analysis results of different datasets may have certain differences (Ying et al., 2020). The gene expression in different samples may be different (Bao et al., 2021). To avoid this problem, integrating multiple datasets and a large number of samples help obtain more solid results (Kou et al., 2020). In this study, we integrated DEGs from two datasets to obtain common DEGs.

**FIGURE 2:**
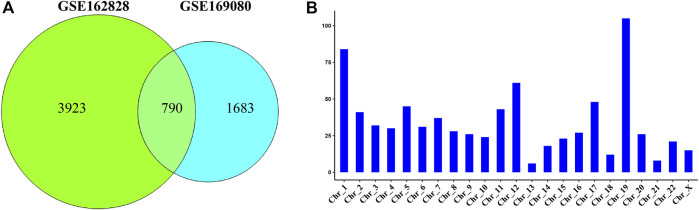
Common differentially expressed genes. **(A)** Venn plot showed the intersection of DEGs in two datasets **(B)** The histogram showed the distribution of DEGs on chromosomes.

The Venn diagram showed that 790 DEGs were shared between the two datasets (Figure 2A). They accounted for 16.8% (GSE162828) and 31.9% (GSE169080) of the two datasets, respectively. There were 3,923 DEGs only in the GSE162828 dataset, and 1,683 DEGs only in GSE169080 dataset. This may be caused by different sequencing technologies and sample heterogeneity.

We defined these 790 DEGs as common DEGs. To further explore the distribution of common DEGs on the chromosomes, we had made statistics on the chromosomal locations of these genes. As shown in Figure 2B, we found that these genes were distributed on every chromosome. Most of these genes were distributed on chromosome 19. On the contrary, they were only 6 DEGs on chromosome 13.

### Analysis of Common Differentially Expressed Genes on Protein-Protein Interaction Network

**FIGURE 3:**
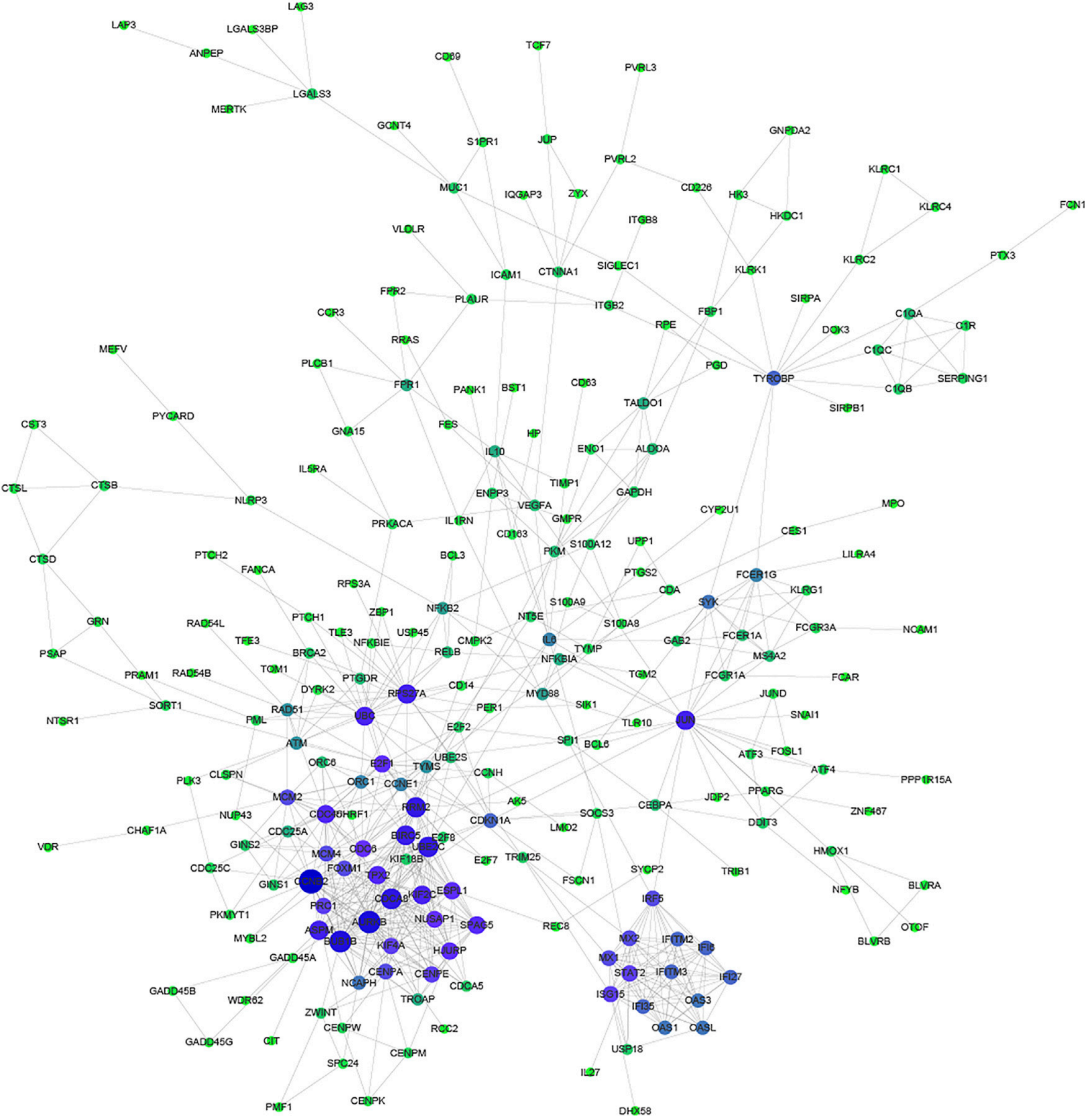
Protein-Protein Interaction network of common DEGs. The size and color of the node depending on the degree, the larger the degree, the larger the node.

Proteins usually perform biological functions in concert. It has been shown that there is a close relationship between Protein-Protein Interaction (PPI) and the biological functions of gene/protein clusters (Li H. et al., 2019). To further analyze the correlation between common DEGs, STRING and Cytoscape were used to construct the PPI network (Figure 3). Part of common DEGs was predicted to have a strong association with other genes. The size and color of the node depending on the degree, the larger the degree, the larger the node.

Especially, *CCNB2*, *CDCA8*, *AURKB*, *BUB1B*, *RRM2*, *BIRC5*, and *UBE2C* had the largest degree. *CCNB2* is an essential component of the cell cycle regulatory machinery (Takashima et al., 2014; Li R. et al., 2019). *CDCA8* is an essential regulator of mitosis and cell division (Zhang et al., 2020). *AURKB* participates in the regulation of alignment and segregation of chromosomes during mitosis and meiosis through association with microtubules (Ahmed et al., 2021). *BUB1B* encodes a kinase involved in the spindle checkpoint function (Zhang et al., 2021). *RRM2* encodes one of two non-identical subunits for ribonucleotide reductase (Mazzu et al., 2020). *BIRC5* encodes negative regulatory proteins that prevent apoptotic cell death (Adamopoulos et al., 2021). *UBE2C* is required for the destruction of mitotic cyclins and cell cycle progression (Jin et al., 2020).

### Functional Enrichment Analysis of Common Differentially Expressed Genes

**FIGURE 4:**
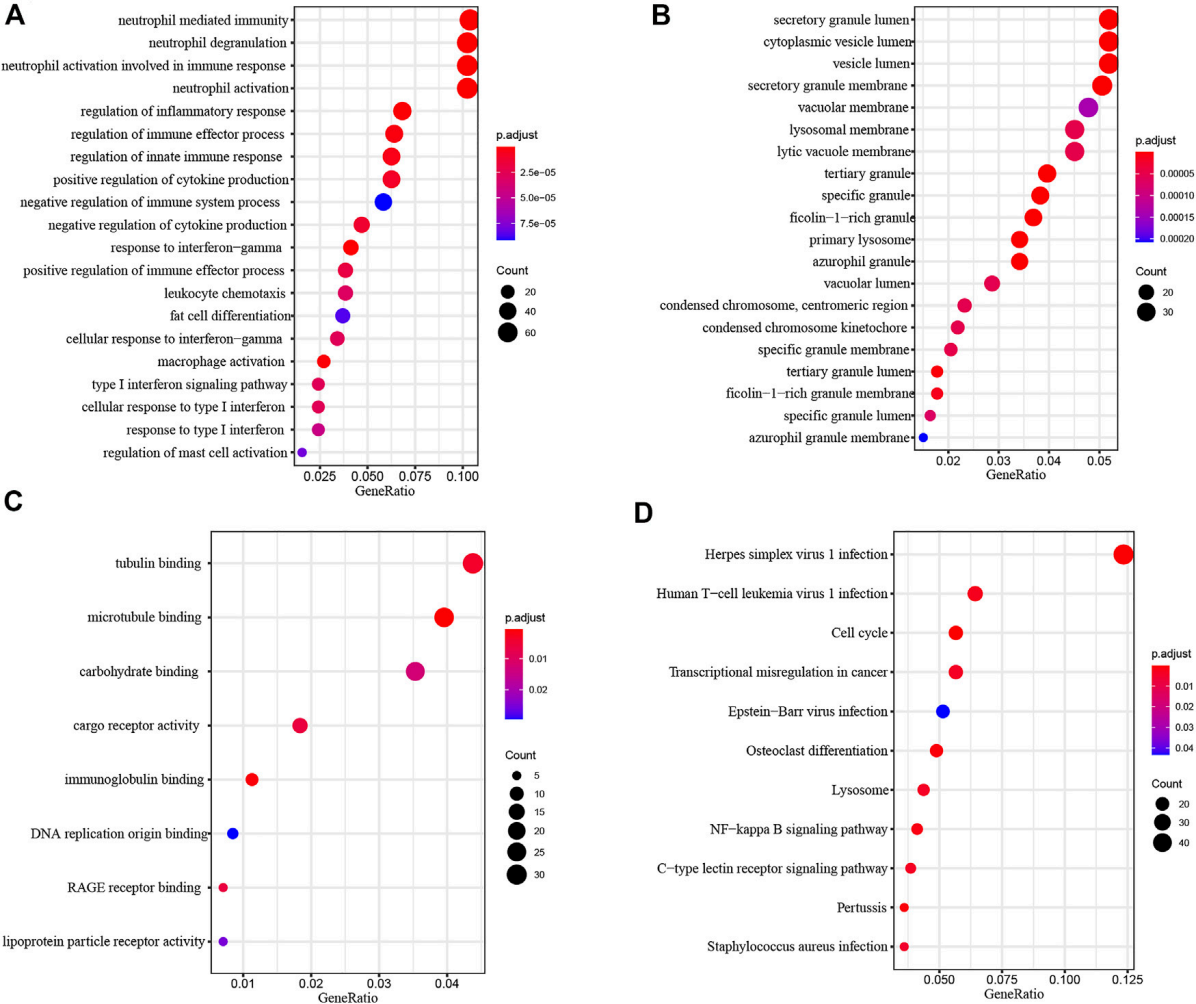
Functional Enrichment Analysis of common DEGs. **(A)** Biological process analysis of common DEGs. **(B)** Cellular component analysis of common DEGs. **(C)** Molecular function analysis of common DEGs. **(D)** KEGG analysis of common DEGs.

To investigate the biological function of common DEGs, we used clusterProfiler to perform Functional enrichment analysis. Biological Process (BP) enrichment showed that the common DEGs were enriched in neutrophil mediated immunity, neutrophil degranulation, neutrophil activation involved in immune response, neutrophil activation and regulation of inflammatory response (Figure 4A). Cellular Component (CC) enrichment showed that the common DEGs were mainly enriched in secretory granule lumen, cytoplasmic vesicle lumen, vesicle lumen, secretory granule membrane and vacuolar membrane (Figure 4B). Molecular Function (MF) enrichment showed that the common DEGs were significantly enriched in tubulin binding, microtubule binding, carbohydrate binding, cargo receptor activity and immunoglobulin binding (Figure 4C).

KEGG pathway analysis provided a potential functional cluster of common DEGs, indicating that the common DEGs were clustered in Herpes simplex virus one infection, Human T−cell leukemia virus one infection, Cell cycle, Transcriptional misregulation in cancer and Epstein−Barr virus infection (Figure 4D).

## Discussion

SLE is a multi-system autoimmune inflammation that can affect multiple organs and cause extensive and severe clinical manifestations (Wu et al., 2021). The current understanding of the pathogenesis of SLE is not comprehensive. The key driving factors involved in the occurrence and development of SLE remain to be determined. In this study, we provided new insights into the transcriptome of SLE based on RNA-seq data.

The results showed that compared with the normal healthy control groups, a large number of genes in SLE patients were abnormally expressed. Through integrated analysis, we found that there were 790 shared DEGs in the two databases. The results indicated that these common DEGs may lead to the occurrence and development of SLE. Previous studies had shown that lncRNA and circRNA are important factors leading to the occurrence of SLE (Cheng et al., 2021; Jiang et al., 2021). We found that the differential expression of these common DEGs might play an important role in this process.

Through further analysis, we found that the DEGs tended to up-regulated in the two datasets. Through protein-protein interaction network analysis of commonly dysregulated genes, we found that there was a strong correlation between these genes. These PPI networks may have affected the occurrence and development of SLE. Pathway enrichment results showed that common DEGs were significantly enriched in immune-related pathways such as neutrophil mediated immunity, neutrophil degranulation, neutrophil activation involved in the immune response.

In summary, we integrated and analyzed high-throughput sequencing RNA-seq datasets to uncover potential molecular mechanisms of SLE. Our findings provide new clues for possible targeted therapy of SLE. Further studies on the functions of those common DEGs hoped to better understand SLE by integrating more data.

## Data Availability

Publicly available datasets were analyzed in this study. This data can be found here: https://www.ncbi.nlm.nih.gov/geo/ GSE162828 and GSE169080
